# In Vitro Activation Early Follicles: From the Basic Science to the Clinical Perspectives

**DOI:** 10.3390/ijms22073785

**Published:** 2021-04-06

**Authors:** Kim Cat Tuyen Vo, Kazuhiro Kawamura

**Affiliations:** Department of Obstetrics and Gynecology, School of Medicine, International University of Health and Welfare, Narita, Chiba 286-8686, Japan; votuyen9788@gmail.com

**Keywords:** in vitro activation, hippo signaling pathway, PI3K/Akt/FOXO3 pathway

## Abstract

Development of early follicles, especially the activation of primordial follicles, is strictly modulated by a network of signaling pathways. Recent advance in ovarian physiology has been allowed the development of several therapies to improve reproductive outcomes by manipulating early folliculogenesis. Among these, in vitro activation (IVA) has been recently developed to extend the possibility of achieving genetically related offspring for patients with premature ovarian insufficiency and ovarian dysfunction. This method was established based on basic science studies of the intraovarian signaling pathways: the phosphoinositide 3-kinase (PI3K)/Akt and the Hippo signaling pathways. These two pathways were found to play crucial roles in folliculogenesis from the primordial follicle to the early antral follicle. Following the results of rodent experiments, IVA was implemented in clinical practice. There have been multiple recorded live births and ongoing pregnancies. Further investigations are essential to confirm the efficacy and safety of IVA before used widely in clinics. This review aimed to summarize the published literature on IVA and provide future perspectives for its improvement.

## 1. Introduction

The majority of achievements in medical science practice are based on basic scientific research. Improved understanding of reproductive physiology has also allowed remarkable advances in artificial reproductive technologies, giving a higher chance to achieve parenthood for millions of infertile couples [1].

As a consequence of global modernization, a higher number of advanced aged infertile women with diminished ovarian reserve (DOR) have been diagnosed in the last decades [2,3]. Due to innovations in oncological treatment, the number of cancer survivors at the reproductive age has been increasing, leading to a higher prevalence of premature ovarian insufficiency (POI) [4]. In addition, there is an increasing necessity for fertility preservation (FP).

During last decades, oocyte donation which cannot fulfill wishes of patients to give birth to genetically related offspring is often the option for patients with DOR and POI. To expand reproductive possibilities to DOR and POI patients, considerable efforts to investigate molecular mechanisms underlying folliculogenesis, leading to a variety of new approaches including follicle regeneration, rejuvenation, and activation [5].

Among these, the activation of early follicles (EFs) including primordial, primary, secondary, and early antral follicles has been recently achieved. For developing the efficient activation system, many basic studies using the animal-model were conducted to identify the signaling pathways governing the activation of early follicles.

IVA has been recently introduced and gradually implemented in clinical practice. This innovation was established from numerous animal experiments, including genetic manipulation studies illustrating the molecular mechanism of two involving signaling pathways in folliculogenesis. The first one is the PI3K/Akt/forkhead box O3 (FOXO3) pathway, which has a crucial role in the activation of primordial follicles (PFs) [6,7,8,9]. The other one is the Hippo signaling pathway which has been recently illustrated to modulate the progress of follicles from secondary to antral stage [10,11]. Based on results obtained from animal-model and in vitro experiments, IVA was implemented to treat POI and DOR women. Healthy babies and other encouraging outcomes have been reported from different groups [11,12,13,14,15,16,17]. Furthermore, several studies suggested the correlation between Hippo pathway’s genes abnormality and polycystic ovary syndrome (PCOS) [18,19,20], leading to a possibility to ameliorate reproductive outcomes of PCOS patients by manipulating the Hippo signaling pathway [21].

To provide a full overview of basic science related to IVA to the scientific community, this review summarized published works from basic scientific studies to clinical reports illustrating the molecular mechanisms and recorded outcomes of this treatment.

## 2. The Activation of the EFs In Vitro

**Figure 1:**
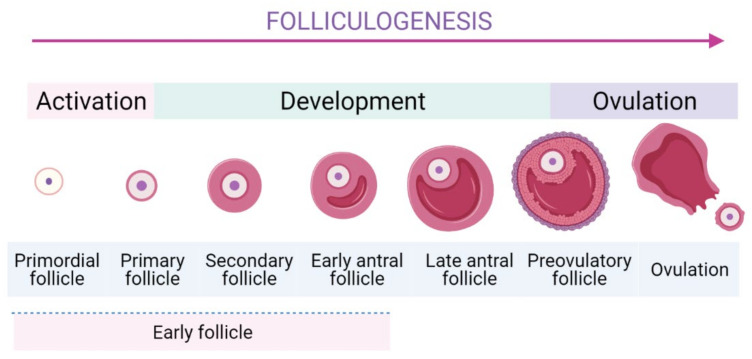
The schematic folliculogenesis from primordial to ovulatory stages.

Folliculogenesis is a complicated process which is generally divided into two stages: the first one is early stage from the PFs to the early antral follicles stage, and the latter one is the early antral follicles to ovulatory stage (Figure 1) [22]. In mammalian ovaries, EFs are the largest population comprising of the PFs, primary follicles, secondary ones, and early antral follicles.

To form the growing follicles, a small number of PFs are cyclically and periodically recruited in the growing pool. The most specific feature of PFs is their long-term dormancy and survival for several decades, ensuring the longevity for female reproductive possibility. Thus, they represent a finite source of ovarian follicles and their recruitment is an indispensable step as it determines reproductive lifespan [23,24]. The activation of EFs is modulated strictly to ensure only a small number of PFs can be activated [10,25]. Subsequently, they undergo continuous folliculogenesis to develop to later stages of development until the ovulation occurs. In PF, a dormant oocyte is enveloped by a single layer of flattened granulosa cells (GCs). In response to stimulators of PI3K/Akt pathway, mTORC1 in GCs is activated [26] and then GCs start to proliferate, cuboidalize, and exclude SMADs proteins [26,27]. These dynamic changes are important signals to initiate the activation of PFs [27]. Moreover, several genes expressed in the GCs were identified to promote the PFs’ activation [26,28]. The internal communication between oocyte and GCs is crucial during follicle development [26].

Although the number of PFs decreases gradually through apoptosis and recruitment, the pool of PFs is not completely exhausted even at the age of menopause [24]. Additionally, it is reported that three out of four POI patients have residual dormant PFs remaining in their ovaries [29]. PFs are the ideal population for PF as they could resist chemotherapies and are well-preserved during cryopreservation [30]. Therefore, consideration to develop an in vitro approach to control the activation of PFs has attracted extensive interest in the past decades [24]. Recently, a number of innovative approaches have been suggested to promote the EFs’ development by using key agents to imitate the physiology ovarian environment.

The first in vitro experiment of PFs was conducted on mice by culturing the whole intact ovaries in serum-containing medium. The spontaneous activation of a small number of PFs in the medulla region, which is similar to the first wave of physiologic PFs activation, was noted. Additionally, the addition of epidermal growth factor (EGF) could enhance the follicle recovery [31]. In bovine and ovine species, culturing ovarian cortex with serum-free medium was also found to activate the PFs [32,33,34]. In human ovary, PFs activated spontaneously in the cultured ovarian cortex regardless of the presence of serum [35,36,37]. The two-step culture procedure using activin comprised of ovarian cortical strips culture in human followed by isolation and culture of the acquired secondary follicles was reported to yield antral stage follicles from the PFs. However, as the timing of follicle growth in this system is highly irregular, the finding has yet to be repeated [37].

These findings suggested that the PFs could be suppressed under the physiological environment, or there are in vitro factors stimulating the PF activation [9]. Although the number of activated PFs was limited in these studies, these encouraging findings placed the important foundation for the development of the activation of EFs in vitro controlled environment. Subsequently, accumulated understanding in mechanisms modulating the activation of EFs has been achieved, leading to a step closer to its potential clinical applicability. The activation of EFs is strictly governed by a number of signaling pathways and components as discussed in the following sections.

## 3. The PI3K/Akt Pathway—The Major Pathway Governing the Activation of PFs

### 3.1. The Cascade of Components of the PI3K/Akt Pathway

The importance of PI3K/Akt pathway in primordial follicle activation has been well-established [6,7,8,9]. This pathway has been shown to be modulated by numerous intraovarian growth factors including Kit ligand (KL), insulin-like growth factor I, platelet-derived growth factor, and EGF [7,38,39]. On the other hand, phosphatase and tensin homolog deleted on chromosome 10 (PTEN), a lipid phosphatase converting phosphatidylinositol-3,4,5-triphosphate (PIP3) back to phosphatidylinositol biphosphate (PIP2), negatively regulates the PI3K/Akt pathway [6]. In transgenic mice with constitutive PI3K activity, a number of PFs remained dormant in postpubertal mice’s ovaries. These dormant PFs showed an accumulation of PTEN in the oocyte nucleus, suggesting that PTEN is the dominant factor in the maintenance dormant state of PFs [40]. Additionally, anti-Müllerian hormone (AMH) was also described to have inhibitory action to the PFs [41]. In mice, incubation with AMH was showed to suppress PFs activation by KL and fibroblast growth factors (FGF) treatments [42].

**Figure 2:**
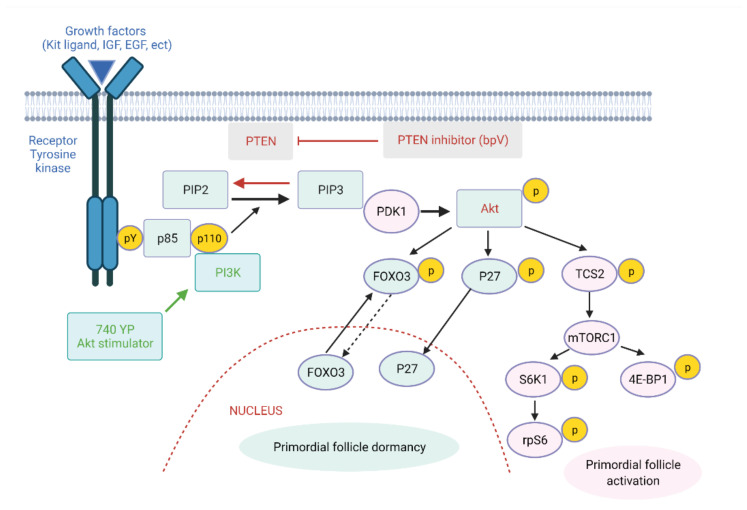
The phosphoinositide 3-kinase (PI3K)/Akt/ forkhead box O3 (FOXO3) pathway in oocytes regulates PF activation. Upon growth factors binding to tyrosine kinase receptors (Kit ligand, insulin-like growth factor (IGF), epidermal growth factor (EGF), etc.), the autophosphorylation of intracellular regions of these receptors takes place. Activated receptors then stimulate PI3K activity, leading to increases in phosphatidylinositol-3,4,5-triphosphate (PIP3) levels and phosphatidylinositol-dependent kinase 1 (PDK1) as well as Akt stimulation. Activated Akt then migrates to the cell nucleus and suppresses FOXO3 actions to promote primordial follicle growth [8,10]. Akt also promotes the phosphorylation of tuberous sclerosis 2 (TCS2), which enhances mammalian target of rapamycin complex (mTORC1) activation. S6 kinase (S6K), ribosomal protein S6 (rpS6) and eukaryotic translation initiation factor 4E binding protein 1 (4E-BP1) are the downstream substrates of mTORC1 which promote the translation of related mRNAs following by cell growth and proliferation.

This pathway is the cascade of signaling pathway with several components (Figure 2). When the KL binds to the cognate tyrosine kinase receptor (c-kit), the phosphorylation of the intracellular region of c-kit enhances the activity of PI3K. PI3K is a lipid kinase comprised of two regulatory subunits (p85 and p110). Once c-kit is activated, these subunits interact with the c-kit, leading to the phosphorylation of the 3-OH group of the inositol ring of inositol phospholipids, transforming PIP2 into PIP3. Later on, PIP3 enhances the activity of phosphatidylinositol-dependent kinase 1 (PDK1), which then serves as a second messenger to phosphorylate Akt, the principal downstream molecule of this pathway [43]. Subsequently, Akt phosphorylates FOXO family including FOXO3, leading to its translocation from the nucleus to the cytoplasm [7,9,43,44]. This relocalization downregulates FOXO3′s activity as a suppressor maintaining the PFs dormancy [7,45]. In addition, Akt phosphorylates tuberous sclerosis 2 (TSC2). Consequently, the inactivation of TSC1/TSC2 complex caused by phosphorylation of TSC2 induces mammalian target of rapamycin complex (mTORC1), another key mediator of PI3K/Akt pathway [43,46,47]. The substrates of mTORC1 such as p70 S6 kinase (S6K), ribosomal protein S6 (rpS6), and eukaryotic translation initiation factor 4E binding protein 1 (4E-BP1) promote cell growth by modulating the protein translation [7]. Moreover, mTORC1–S6K1–rpS6 contributes to the survival of PFs [48]. Additionally, TSC1 was indicated to interact with PTEN and suppress the mTORC in the activation of PFs [49].

### 3.2. The Genetic Evidence Demonstrating the Roles of PI3K/Akt Pathway in Folliculogenesis

Using genetically modified mouse models, a number of studies illustrated the crucial roles of the PI3K/Akt pathway in the activation of PFs. The deletion of *PTEN* in oocytes in mice resulted in an extensive and precocious activation of PFs [48,50]. In addition, mutant mice with specific deletion of *PTEN* led to increased granulosa cell proliferation, and ovulatory efficacy as well as to decreases in follicle atresia [51]. The majority of PFs in mice lacking *Pdk1* experienced an early depletion at dormant state, leading to POI phenotype in early adulthood [48]. Similarly, in mice with genetic ablation of *Foxo3a*, a higher rate of primordial follicle activation was noted, leading to early infertility [52,53]. The deletion of *TSC1* or *TSC2* increases protein translation in oocytes, resulting in follicular overactivation [54,55]. Of note, in mice lacking both *PTEN* and *TSC1*, an enhancement of oocyte growth was observed. Additionally, the loss of mTORC1 signaling in oocytes stimulates the PI3K signaling cascade to maintain normal follicular growth [54,56]. These results suggested that mTORC1 signaling is an independent pathway that also controls follicular activation. Meanwhile, in mice lacking *PTEN, FOXO3* was constantly phosphorylated and suppressed [50]. The deletion of both *PTEN* and *FOXO3* resulted in genetic equivalence to specific deletion of *PTEN*, indicating the dependent role FOXO3 from PI3K pathway in PFs’ activation [45].

### 3.3. Stimulation of the PI3K/Akt Pathway in the Mammalian Ovary

Several experiments were carried out in animal models to clarify the roles of PI3K/Akt pathway in PFs’ activation. In three-day-aged mice, ovaries treated with 100 μM bpV(HOpic) (a bisperoxovanadium inhibitor of protein phosphotyrosine phosphatases with selectivity for PTEN) plus 740YP (a PI3K activator) for 48 h were dramatically larger with higher number of follicles at antral stages than control ovaries. Mature oocytes could be retrieved and fertilized after in vitro fertilization (IVF), resulting in live birth of healthy pups [57]. Another study incubated neonatal mice’s ovaries in bpV(HOpic) (1 µM) for 24 h followed by the transplantation to kidney capsule of paired ovaries (treated and untreated). The phosphorylation of TSC2 was elevated in treated oocytes, indicating the enhanced activity of PI3K/Akt signaling in the bpV(HOpic) treated ovaries. In comparison to the control ovaries, a higher number of follicles at the preovulatory stage was found in bpV(HOpic)-incubated ovaries and slightly higher numbers of healthy live pups were derived from the treated ovaries. The authors also revealed that short-term incubation of PTEN inhibitor neither lead to any tumor formation in the graft or in the recipient mice nor affected the development of already growing follicles [58]. The mTOR activator (MHY1485) was also demonstrated to promote the EFs’ development in ten-day-old mice. Co-treatment with MHY1485 and Akt stimulators further increased the number of antral follicles and decreased the number of PFs compared to treatment with only Akt stimulators [47]. Similarly, the incubation combining both Akt and mTOR stimulators (phosphatidic acid or propanolol) of ovarian samples of neonatal mice and human demonstrated synergetic effects on follicular development. The combination resulted in more developed secondary follicles and fewer degenerated follicles. After IVF treatment using retrieved oocytes, the number of 2-cell embryos obtained from the treated group was higher than the control group, resulting in 33 healthy pups [59]. In consequence, the authors proposed in vitro transient treatment with mTOR and PI3K activators to treat women with low follicle reserve [59].

In swine ovarian tissue incubated with bpV(HOpic) (1 μM) for 48 h, the number of growing follicles was significantly higher compared to the control ovaries with a higher percentage of growing follicles. The degeneration rate in the incubated follicle population was approximately 30%, which was not different between bpV(HOpic) and control groups [60]. In ovine, the combined treatment with 15 μM bpV and 100 ng/mL stem cell factor (SCF) was the most effective strategy for the activation as well as minimizing degeneration rate of PFs. The tissue fragments treated with 15 μM bpV had the higher expression of genes involved in the development of primary follicles accompanied by the lower apoptosis-related genes compared to control groups [61]. In a recent study, peroxisome proliferator-activated receptor gamma (PPARγ) was demonstrated to activate murine PFs via the PI3K/Akt pathway. In mice treated with PPARγ modulators, there were a decline in PTEN levels and increased nuclear exclusion of FOXO3a in PFs, suggesting the stimulation of PI3K/Akt pathway. The treated ovarian transplanted graft showed a higher number of mature oocytes and the oocytes could fertilize to deliver normal live pups [62]. The recent experiment suggested that EGF could be an alternative agent to activate the primordial follicle through elevating CDC42-PI3K signaling and PI3K/Akt pathway in both murine and human ovarian tissue. After treatment of mouse ovaries with EGF by in vitro culture together with in vivo topical administration of EGF dissolved in the Matrigel into ovarian bursa, the histological results demonstrated an increase in the percentage of growing follicles in EGF-treated ovaries compared to controls [39]. In human tissues, similar results were observed by culturing ovarian cortical fragments with EGF [39].

Some authors raised concerns about the impact of manipulation of PI3K/Akt on the follicular function and survival [44], especially DNA damage after PI3K/Akt pathway stimulation. Bovine ovaries incubated with 1 and 10 μM bpV(HOpic) for 24 h had increased activation of the primordial follicle suggested by considerably higher proportion of growing follicles in the treated tissues [63]. However, the exposure to bpV(HOpic) was found to be associated with DNA damage and impaired DNA repair competence, especially at the high dose of bpV(HOpic) in GCs [63]. The majority of the follicles under high dose bpV(HOpic) showed apoptosis in GCs after 6 days of culture. It was hypothesized to be the consequence of high metabolic activity in GCs after the activation. It should be noted that the proportion of morphologically normal follicles in the low dose group and control group were not different, reflecting the dose-effect of bpV(Hopic) in the DNA damage [63]. Another explanation for this result is the compromised contact between the somatic cells and oocyte [64]. In transgenic mice, hyperactivity of the PI3K/Akt pathway in the perinatal oocytes led to lack of coordination between oocyte and GCs’ growth, resulting in enlargement of follicle size and anovulation, but oocytes were meiotically competent [40]. In another study, sirtuin 1 synergistically enhanced the activation of GCs and oocyte via mTOR signaling pathway in GCs and both PI3K and mTOR pathways in oocytes, suggesting its potential for improvement of coordination between oocyte and GCs growth [65]. To minimize damage to follicles, recent work suggested that short exposure to mTORC1 inhibitor could improve follicle survival and steroidogenesis [66].

In human ovary, after incubation with 1 µM bpV(HOpic) for 24 h, the number of growing follicles in the bpV(HOpic)-exposed ovarian tissues increased remarkably. However, the secondary follicles isolated from the exposed ovaries showed a lower survival rate when comparing to the control group [67]. Of note, despite low dose of bpV(HOpic), the ovarian tissues were further incubated for another five days with half the medium being removed. Such long-day incubation could interfere with the survival of follicles as supposed in some recent comments [5]. However, in a subsequent experiment, incubation with 100 µM bpV(HOpic) for 25 h was shown to increase the percentage of growing follicles. The follicular viability of the activated group and control group was not significantly different [68]. In more recent experiment, the ovarian tissues cultured with 150 μg/mL 740YP and 30 μM bpVHOpic for 48 h exhibited a high proportion of activated follicles, leading to an increased transitory follicle population. Meanwhile, culture with mTORC1 inhibitor had a lower number of growing follicles throughout the culture period, indicating the suppressive effect on the follicular activation. Notably, follicular survival in these two cultured groups was not different from the control group during culture, indicating the safety for manipulating the PI3K/Akt pathway upon the viability of follicles [66].

## 4. The Hippo Signaling Pathway—Another Signaling Pathway Regulating the Growth of EFs

### 4.1. The Mechanism of Hippo Signaling Pathway

The second major pathway in IVA is the Hippo signaling pathway, which has been described as a fundamental pathway regulating mammalian organ size [69,70,71]. This pathway is regulated by several upstream components involved in cell adhesion, shape, and polarity [72]. Among these components, actin acts as a multifunctional protein that forms microfilaments to maintain important cellular processes. Once globular actin (G-actin) polymerizes to form the filamentous pattern (F-actin) in the stress fiber, the Hippo signaling pathway is disrupted [73].

**Figure 3:**
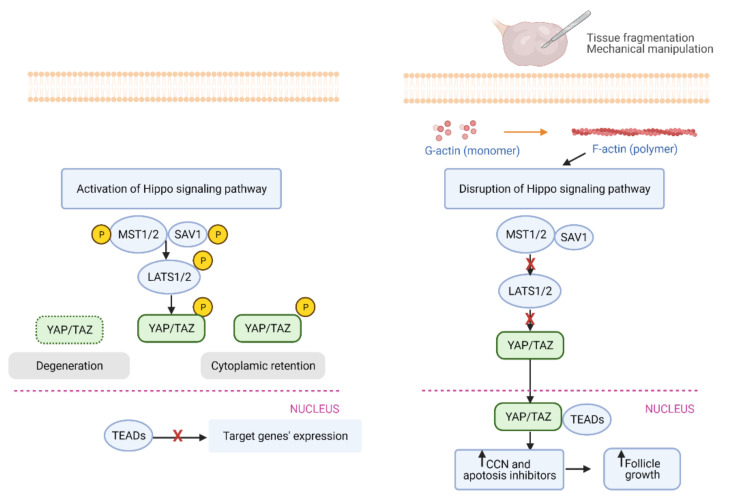
Tissue fragmentation and mechanical manipulation disrupt ovarian Hippo signaling pathway and promote follicle growth. (**Left**) Without tissue fragmentation and mechanical manipulation, Hippo signaling is activated. Kinase complexes consisting of the mammalian sterile-20 like serine/threonine kinase 1/2 (MST 1/2) and Salvador (SAV) are phosphorylated. Subsequently, large tumor suppressor 1/2 (LATS1/2) phosphorylates Yes-associated protein (YAP)/ transcriptional co-activator PDZ-binding motif (TAZ), leading to the degradation of YAP/TAZ or retention of YAP/TAZ in the cytoplasm. Consequently, transcriptional enhanced associate domains (TEADs) cannot promote the expression of target genes. (**Right**) Ovarian fragmentation leads to actin polymerization, resulting in nuclear translocation of YAP. Nuclear YAP interacted with TEADs to increase the expression CCN growth factors and baculoviral inhibitors of apoptosis repeat containing (BIRC) apoptosis inhibitors, resulting in follicle growth.

This pathway controls cell proliferation and organ growth via the kinase complexes consisting of the mammalian sterile-20 like serine/threonine kinase 1/2 (MST 1/2) and Salvador (SAV) as well as the large tumor suppressor 1/2 (LATS 1/2). These components suppress the Yes-associated protein (YAP) and transcriptional co-activator PDZ-binding motif (TAZ) through phosphorylation and cytoplasmic retention. The disruption of Hippo signaling pathway decreases phosphorylation of YAP, leading to increased nuclear YAP levels [74]. Upon binding of YAP to TEAD (Transcription factors containing the TEA/ATTS DNA binding domain) transcriptional factors, the expression of CCN growth factors and baculoviral inhibitors of apoptosis repeat containing (BIRC) are increased. Eventually, these factors stimulate cell growth, survival, and proliferation (Figure 3) [69,74,75].

In the mammalian ovary, the fragmentation of ovarian cortex into small cubes was found to disrupt the Hippo signaling pathway by increasing the polymerization of G-actin into F-actin. Consequently, increases in YAP nuclear translocation stimulated the expression of CCN growth factors and BIRC apoptosis inhibitors, resulting eventually in secondary follicle growth [10,11] (Figure 3).

### 4.2. Basic Studies Demonstrating the Role of Hippo Signaling Pathway

Several studies described the mechanism and importance of this pathway on folliculogenesis. In ten-day-aged mice, after one hour of ovarian fragmentation, the ratios of F-actin to G-actin increased transiently and decreased pYAP to total YAP ratios were observed, showing disruption of the Hippo pathway. The ovarian cubes were then grafted under hosts’ kidney capsules, which is the typical site for grafting tissues because of its high vascularity [76]. After five days, ovaries with mechanical manipulation were remarkably larger with higher percentages of late secondary and antral/preovulatory follicles in comparison to paired intact ovaries. Similarly, incubation of juvenile mice ovaries with jasplakinolide, a converter of G-actin to F-actin or sphingosine-1-phosphate (S1P), a suppressor of Hippo signaling pathway, for 30 min also increased nuclear YAP and subsequent CCN2 transcript levels, resulting in the stimulation of follicle growth [77]. Moreover, the addition of S1P to the culture medium reduced follicle atresia and improved the primordial follicle quality in the human ovary [78,79,80].

Many other findings indicate the importance of Hippo signaling pathway in EFs’ development. MST1/2, LATS1/2, YAP, and TAZ are expressed in different development stages of follicles in mouse as well as human ovaries [11,81,82]. Recent computational data also noted that the YAP/TAZ signaling pathway is active in vivo [83]. Moreover, the collagen-rich ovarian cortex conferring a rigid physical environment was suggested to be crucial to maintain the oocyte dormancy. Similar to the ovarian fragmentation, the enzymes degrading the extracellular matrix secreted by GCs were found to activate the PFs [23,84]. The digestion of the matrix by collagenase type IV induced the nuclear export of FOXO3 and oocyte growth [23]. A recent study reported that deposition and remodeling of mechanical matrisome components (collagen, elastin, elastin microfibril interface-located protein 1 [EMILIN-1], fibrillin-1, and glycosaminoglycans [GAGs]) was associated with the EFs’ activation [85]. These findings pave the way for possible therapeutic targets at the extracellular matrix level, such as mechanical stimulation or antifibrotic treatments, to activate the EFs.

A recent study revealed that culturing the intact neonatal mice ovaries without Hippo or PI3K/Akt modulators could also increase the CCN2 expression and the KL staining. However, sectioned ovaries resulted in a remarkable increase in follicular counts compared to the intact ovaries and fresh ovaries, but with a higher rate of cell death [30]. The method of ovarian fragmentation was proposed to contribute to the variable findings among studies [67].

Genetic evidence also illustrated the crucial role of Hippo signaling pathway in follicle growth. *LATS1* null mice presented with a POI phenotype because of an early extensive follicular activation [86]. *YAP*-knockout mice had a smaller size as well as the number of growing follicles, accompanied by a higher number of atretic follicles compared to control [87]. In the human ovary, YAP protein was mainly localized to the nuclei of GCs from the primary to the preovulatory stage [87]. Deletion of *CCN* exhibited a decrease in the number of preantral follicles in mice [88]. In humans, deletion of suppressing actin depolymerization genes and other related Hippo pathways were identified in subfertile or infertile women [88,89,90].

### 4.3. The Interaction between Hippo and PI3K/Akt Signaling Pathways in EFs’ Activation

Some studies indicated that the Hippo signaling pathway works synergistically with PI3K/Akt activators to accelerate primordial follicle recruitment [30,66,91]. By combining Hippo disruption with the Akt stimulating drugs, a higher number of late and antral follicles was observed. Mechanical manipulated grafts had a 3.1-fold higher number of retrievable oocytes compared to intact grafts. Several healthy pups were achieved after IVF and embryo transfer procedure [11]. More recently published study suggested that Hippo pathway, acting downstream of Akt signaling, also regulates primordial follicular activation [82]. *YAP1* knockout mice had a considerable increase in the number PFs accompanied by the decline of primary follicles [82]. Notably, ovarian fragmentation increased the expression of not only YAP1 but also Akt and rpS6 [66]. In addition, the mTORC1 inhibitor showed a suppressive action on both Hippo and PI3K/Akt pathways [30]. In human ovarian tissue, one study reported that ovarian fragmentation upgraded immediately BIRC1 and CCN2, increasing follicular growth near the cutting site [66]. Additionally, the combination of both fragmentation and incubation with 740YP and bpV(HOpic) generated a second wave of BIRC1 and CCN2 expression, concomitant with high expression of KL and Akt [66]. These findings suggested the interaction between these two pathways in follicle growth, similar to other studies conducted in different cell types [92,93]. Consistent with these findings, a recently published study indicated that fragmentation increased the number of secondary follicles in oncological patients [94].

## 5. Other Regulatory Components Stimulating the EFs’ Development

The development of EFs is a complicated process governed by a number of signaling pathways and components [25,95,96]. Recent computational studies revealed that these components interact in different ways to promote the transition from PFs to the primary stage [83]. Additionally, it is reported that more than 1000 genes are involved in EFs’ survival and development [25,95,97]. These findings suggested that there should be additional possible future therapeutic targets for the IVA approach.

### 5.1. Growth Factors Governing the Early Folliculogenenesis through Autocrine and Paracrine Mechanisms

There are several factors, generated from the oocyte, GCs, stromal cells, neighboring follicles, and local blood vessels, known to enhance the EFs’ development. Among these factors, transforming growth factor beta 1 (TGF-β1) family is considered the most prevalent in EFs’ development. Belonging to this family, growth and differentiation factor 9 (GDF-9) and bone morphogenetic proteins (BMPs) have been intensively investigated. GDF-9 derived from oocytes was described as necessary for EFs for follicular growth from the early PFs to the preantral stage, especially at the transition of primary to secondary follicle stage [98,99]. Similarly, BMPs, another oocyte-secreted factor, enhance the early folliculogenesis [10,99]. In addition, they stimulate the activity of vascular endothelial growth to increase the angiogenesis in theca cells [99], which in turn enhances the nutrition supplement to the GCs and oocyte in EFs for promoting the activation of dormant follicles [26,100,101]. In human ovaries, one study indicated that the addition of GDF-9 and BMP-15 in culture synergistically accelerated the activation of EFs compared to the culture with GDF-9 only [102]. Once binding to their receptors at the GCs, they stimulate the SMAD pathway which in turn activate the PFs in mouse ovary [27]. SMAD3 signaling was represented to regulate genes involved in cell cycle regulation in GCs of PFs [103]. Moreover, GDF-9 and BMP-15 have a protective action to GCs from apoptosis, resulting in better follicular survival [99]. The mutation of their genes resulted in subfertility, abnormal ovulation, and POI in several species [104,105,106]. Activins, other members of TGF-β1 family, have been found to promote the survival of oocytes and the formation of PFs via smad2/smad3 pathway [107]. Culturing bovine ovary with activins promoted the PFs development [108].

Also, neurotrophins (NTs) and glial cell line-derived neurotrophic factor (GDNF) also take part in the assembly and activation of PFs. In murine, culturing newborn ovaries with NTs showed an extensive activation of PFs [109]. In consistence, GDNF increased activation rate of PFs in ovine ovary [110]. In human ovary, when binding to its receptors (Trk), NTs activate several pathways including PI3K/Akt and Mitogen activated protein kinase (MAPK) pathways which, in turn, activate the PFs [111]. The addition of NT4 to the culture medium enhanced PFs’ assembly in fetal ovary [112].

### 5.2. FSH

FSH is generally described as one key gonadotropin modulating the latter stage of folliculogenesis. However, several data suggested that FSH has specific roles in EFs’ development. In mice, gonadotropin-deficiency resulted in a higher number PFs [113]. In another experiment, FSH accelerated the development of PFs in bovine ovary [108]. In primate, FSH treatment prevented the depletion of EFs in freshly-grafted tissues. The EFs with normal morphology were also increased in FSH-treated tissues in comparison with the control ones [114]. Also, the FSH extended the cell metabolism and modulated MAPK signaling pathway in preantral follicles, suggesting certain roles of FSH in early folliculogenesis [115].

### 5.3. Anti-Müllerian Hormone-an Inhibitory Factor for EFs’ Development

AMH, a member of the TGF-β family, is secreted by growing EFs. Unlike other members, AMH has been proposed to inhibit the PFs’ activation and EF’s development [24,64]. In rat ovaries cultured with AMH, PFs kept at the quiescent state despite the presence of other known stimulators (e.g., FGF and KL) [42]. In ovine, knockdown of AMH activity using active immunization against AMH did not affect the PFs recruitment, but decreased the population of preantral and small antral follicles, suggesting its regulation of follicle development until the gonadotropin-responsive phase [116]. However, in pre-pubertal ovine ovaries, the high dose of AMH (at 50 ng/mL or 100 ng/mL) could inhibit PFs activation, suggested by presence of a higher number of PFs and a lower number of intermediate follicles compared to the control and low dose of AMH treatment groups [117]. In bovine, the addition of high doses AMH to the culture medium also suppressed the activation of primordial follicles and growth of activated follicles in fetal ovarian tissues [41]. These findings indicated that the action of AMH on PFs’ activation might vary according to species, dose, and development stage, complicating its preclinical testing for FP.

## 6. Activation of EFs in Current Reproductive Practice

**Figure 4:**
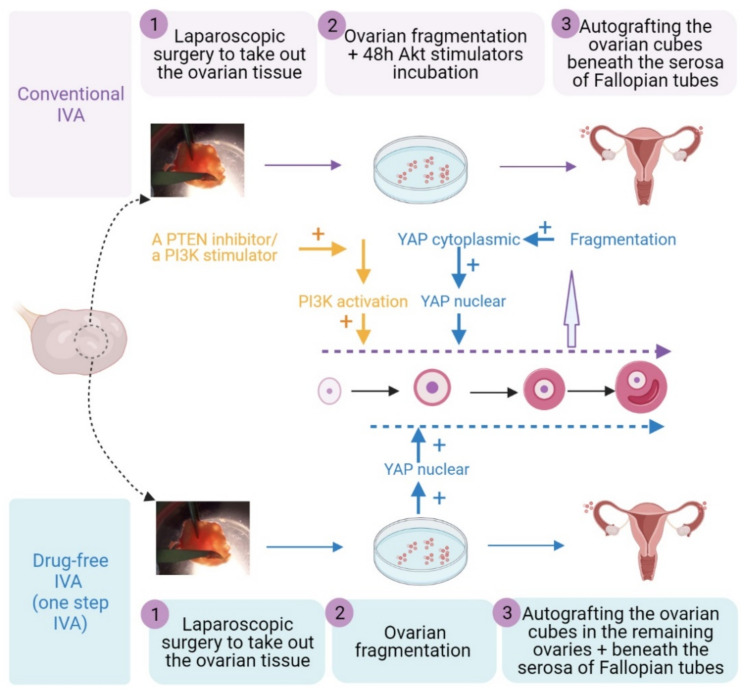
Comparison of the clinical approach between conventional IVA and drug-free IVA. In conventional IVA, one or both ovaries from POI patients were removed under laparoscopic surgery and cut into strips before vitrification. After thawing, strips were fragmented into 1–2 mm cubes, before incubation with Akt stimulators. Two days later, cultured cubes were autografted under second laparoscopic surgery beneath the serosa of Fallopian tubes. In drug-free IVA, the ovarian cortical tissues were removed and fragmented into 1–2 mm cubes followed by grafting back without culture in remaining ovaries and beneath the serosa of Fallopian tubes within the same operation.

Better understandings of the pathways and agents modulating the EFs’ growth could allow the development of therapeutic approaches to improve female fertility competence [24]. Currently, one approach named IVA was introduced to clinical practice around the world to treat POI and DOR patients. In this procedure, ovarian cortex obtained from patients through laparoscopic surgery is fragmented into small cubes (approximately 1–2 mm^3^) to disrupt Hippo signaling. The cubes are incubated with a PTEN inhibitor and/or a PI3K stimulator for 2 days followed by autotransplantation beneath the serosa of the Fallopian tubes [11] (Figure 4). Subsequently, the follicle growth is stimulated by exogenous gonadotropin under suppression of elevated luteinizing hormone to generate competent mature oocytes for subsequent IVF procedure. In the second publication, we reported 2 healthy live births in 20 POI patients who had remaining follicles by histological analysis and the increasing number of unpublished deliveries were presented in scientific conferences [12]. Recently, conventional IVA was modified to develop a one-step procedure named as drug-free IVA [16,118]. In this approach, ovarian cortical tissue is removed under laparoscopic surgery and fragmented into small cubes. The ovarian cubes are immediately grafted back beneath the serosa of Fallopian tubes and into remaining ovaries between cortex and medulla tissues. Growing evidence showed promising outcomes after the IVA treatment in clinical practice. According to our review and personal communications, different authors’ groups published at least a total of 18 healthy live births and three more ongoing pregnancies at the time of publication in POI and DOR patients (POI: two live births [11,12], one live birth [13] and one live birth [14]; DOR: 10 live births [15], one live birth plus two ongoing pregnancies [16], and three live births and one ongoing pregnancy [17]) and more unpublished deliveries and ongoing pregnancies were reported in scientific conferences.

Additionally, IVA could be a beneficial option for oncological patients and other female populations attempting FP as the IVA approach could maximize the number of oocytes in infertile women [119]. Indeed, a recent study reported that fragmentation could activate the PFs through the Hippo signaling pathway, resulting in a higher number of secondary follicles in cancer patients [94]. After cryopreservation of ovarian tissues for FP, the grafting of activated ovarian cubes can be conducted at any time according to their child-bearing wishes of patients after remission of the original disease. IVA can minimize age-related deterioration of oocyte quality in young age patients by the cryopreservation step. Although there has been no reported live birth in cancer patients after IVA, some other positive results were reported. A short-term incubation PTEN inhibitor was demonstrated to accelerate the follicle development without increasing the apoptosis in ovaries obtained from oncological patients [68]. Ovarian fragmentation was demonstrated to increase secondary follicle number in cancer patients by interacting with the Hippo signaling pathway, indicating this pathway’s role in EFs’ growth [94]. Furthermore, the in vitro culture system to generate the mature oocyte from PFs has been suggested to minimize the risk of re-implantation of tumor cells [120,121].

In this approach, the activation of PFs is the initial and most important step as majority of PFs exist in the resting state. By combining with other steps, a recent report achieved the first human oocyte with mature structure derived from an in vitro–grown unilaminar follicles for the PFs [122]. In addition, the prospective view of IVA is applying the follicle isolation technique accompanied with preparation of matrix for supporting follicle growth to minimize the residual malignancy in grafts. On the other hand, some authors questioned the necessity of IVA in FP since several works presented that the activation could occur spontaneously after the conventional FP [123,124,125]. An excessive activation induced follicle loss leading to the reduction of the lifespan’s graft, resulting in an unfavorable outcome in the long-term aspect [126,127]. The current conventional FP was reported to offer pregnancies as long as five years post transplantation [127].

**Figure 5:**
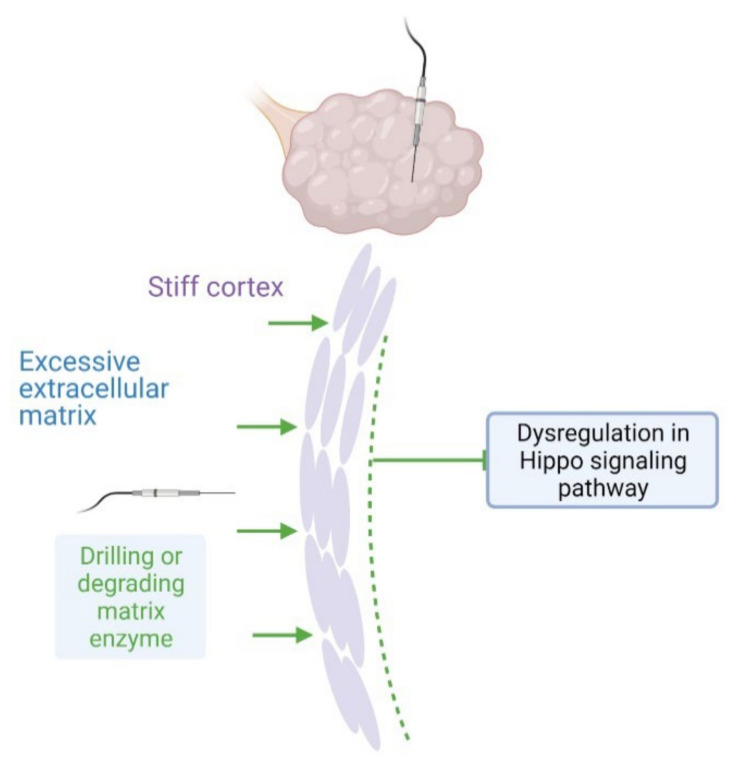
The schematic hypothesis for correlation of polycystic ovary syndrome (PCOS) and Hippo signaling pathway. (1) The mechanical damage and/or degrading enzyme approaches or (2) local administration of the Hippo downstream CCN growth factors and/or actin polymerization drugs for disruption of Hippo signaling aim to ameliorate the dysregulation of Hippo signaling pathway in PCOS patients for resumption of follicle growth.

In addition, several studies revealed the association of the expression of several Hippo signaling pathway genes (*YAP1, MOB1A, MOB1B, and WWTR1*) and PCOS [18,19,20,128]. In PCOS patients, the methylation level of YAP1 promoter region exhibited a significantly lower level than that of the control group [129]. Based on these findings, using CCN growth factors and/or local administration of actin polymerization drugs were suggested as a novel treatment for PCOS [11]. Hippo gene-targeted therapeutics were also supposed to be beneficial on fertility and systemic symptoms for PCOS patients [18] (Figure 5).

However, like other innovations in medical science, further studies are needed to improve this procedure as a successful and safe approach. Because IVA is ineffective in patients without residual follicles, developing a novel method to identify the presence of residual follicles before the IVA surgery can improve the outcome of IVA. Some studies have suggested the optical coherence tomography as imaging techniques for live ovarian imaging to localize the residual follicles [130,131,132]. Using PTEN inhibitor was also reported to provoke destructive effects with various doses and timing [63,67,133]. Further studies are necessary to investigate the most efficient and safe dose and timing of culture. As several other components were found to accelerate the EFs’ activation, developing IVA approach using alternative agents may improve this procedure [96].

## 7. Summary

Numerous researches provided scientific evidence for pathways underlying the IVA approach. IVA offered encouraging outcomes to the poor prognostic infertile women in the clinic. Individualization in treatment is a crucial aspect of clinical practice. In patients with DOR or early stage of POI, drug-free IVA is more beneficial compared to conventional IVA as it decreases the invasiveness of surgical approach and avoids the unfavorable effects of tissue culture on follicles. In terms of FP, IVA should be applied to only patients with low ovarian reserve and the requirement for motherhood achievement is urgent.

## Data Availability

Not applicable.
